# Fast Quantification of Air Pollutants by Mid-Infrared Hyperspectral Imaging and Principal Component Analysis

**DOI:** 10.3390/s21062092

**Published:** 2021-03-17

**Authors:** Juan Meléndez, Guillermo Guarnizo

**Affiliations:** LIR–Infrared Laboratory, Department of Physics, Universidad Carlos III de Madrid, 28911 Leganés, Spain; juan.melendez@uc3m.es

**Keywords:** infrared imaging, multispectral and hyperspectral imaging, air pollution monitoring, remote sensing and sensors, spectroscopy, fourier transform, image processing

## Abstract

An imaging Fourier-transform spectrometer in the mid-infrared (1850–6667 cm${}^{-1}$) has been used to acquire transmittance spectra at a resolution of 1 cm${}^{-1}$ of three atmospheric pollutants with known column densities (Q): methane (258 ppm·m), nitrous oxide (107.5 ppm·m) and propane (215 ppm·m). Values of Q and T have been retrieved by fitting them with theoretical spectra generated with parameters from the HITRAN database, based on a radiometric model that takes into account gas absorption and emission, and the instrument lineshape function. A principal component analysis (PCA) of experimental data has found that two principal components are enough to reconstruct gas spectra with high fidelity. PCA-processed spectra have better signal-to-noise ratio without loss of spatial resolution, improving the uniformity of retrieval. PCA has been used also to speed up retrieval, by pre-calculating simulated spectra for a range of expected Q and T values, applying PCA to them and then comparing the principal components of experimental spectra with those of the simulated ones to find the gas Q and T values. A reduction in calculation time by a factor larger than one thousand is achieved with improved accuracy. Retrieval can be further simplified by obtaining T and Q as quadratic functions of the two first principal components.

## 1. Introduction

Public concern about the adverse health effects of air pollution has increased considerably in recent years. This growing concern is being progressively translated into more restrictive legislation [1]: new emission limit values (ELVs) are set for previously unregulated pollutants, and more stringent levels are established for those already regulated. There is thus an increasing need to develop reliable methods for the measurement of atmospheric gases at immission levels. An example of this trend is the IMPRESS 2 project, funded by the research program EMPIR (European Metrology Programme for Innovation and Research) of the European Association of National Metrology Institutes (EURAMET), with the aim of improving measurement of pollutant gases at several levels: to develop new reference measurement methods for gases not yet regulated, to improve hyperspectral techniques, to determine uncertainty and traceability of mass emission measurements, etc. [2].

Ideally, a measurement method for air pollutants should be both versatile and accurate. Since all pollutant gases show characteristic absorption–emission bands in the infrared (IR) spectral region, IR optical techniques are such a versatile method with the additional advantage of providing remote and non-intrusive measurements. There are many techniques for IR optical gas sensing (see [3] for a comprehensive review) but high resolution spectroscopy is the most wide ranging in its applications, being able to detect several gases at the same time, and has the potential for high accuracy, since the dependence of line intensities on temperature and concentration is very well known.

Due to these features, Fourier transform spectrometry has been used for a long time to measure emissions from smokestack effluents and other industrial sources [4,5,6], but in recent years, imaging spectrometers have conferred additional power to this technique [7]: it has become possible to map column densities *Q* (concentration·path product) of pollutants and plume temperatures *T* [8] over a large area, or to track gas flows and estimate effluent mass flow rates [9]. Cooler sources, such as automobile exhaust emissions, have also been measured in absorption mode [10], as well as ambient-temperature greenhouse emissions [11].

These studies apply techniques originally developed for non-imaging absorption spectroscopy to each pixel of the acquired datacube. It is possible, however, to take advantage of the large amount of data provided by imaging instruments to improve the sensitivity and signal to noise ratio. The objective of this paper is to study the absorption spectroscopy of pollutant gases in the atmosphere in the context of hyperspectral imaging, taking advantage of those possibilities. In particular, the well-known statistical technique of principal component analysis (PCA) is applied to gas spectra in the datacube, first to filter out noise and then to fasten retrieval of T and Q values. A simple radiative model applicable to field measurements is defined, although in this work it has been used only for laboratory measurements with a gas cell in order to evaluate its accuracy for the determination of gas concentrations.

Three gases have been studied: methane (CH${}_{4}$), nitrous oxide (N${}_{2}$O) and propane (C${}_{3}$H${}_{8}$). The first two are greenhouse gases and the third is a hydrocarbon that frequently appears jointly with methane and whose spectral features are in the same spectral region. For each of them, a mixture of known concentration has been prepared, and measured with a hyperspectral imager that operates in the mid-infrared band. Values of T and Q have been retrieved by fitting experimental spectra with simulated ones, and have been compared with the nominal values to assess the accuracy of the method. It has been demonstrated that processing with PCA increases signal to noise ratio which, in turn, improves the accuracy of retrieval, without losing spatial resolution or increasing acquisition time.

The basics of our approach are described in Section 2. After briefly explaining the radiative model in Section 2.1, the retrieval procedure is outlined in Section 2.2 and detailed in Section 2.3. The experimental setup and the measurements performed are described in Section 3. Principal component analysis is exposed and applied to noise filtering of spectra in Section 4; then it is applied, in Section 5, to reduce the dimensionality of spectra, thus making possible a faster retrieval of column density *Q* and temperature *T*. Retrieval is further simplified in Section 5.3 by defining polynomial functions that provide *Q* and *T* directly as functions of the principal components of the spectra. Finally, conclusions are summarized in Section 6.

## 2. Radiative Model and Retrieval Method

Nearly all gas molecules have characteristic absorption/emission spectra in the infrared (IR) spectral region, due to transitions between ro-vibrational levels. For a specific line at wavenumber ν with absorptivity *a*, gas transmittance is given by the Lambert–Beer law:

(1) $$\begin{array}{c} \tau_{g}\left(\nu ,C_{g},T_{g}\right)=e^{-a\left(\nu ,T_{g}\right)C_{g}L_{g}}\equiv e^{-a\left(\nu ,T_{g}\right)Q_{g}} \end{array}$$

where $L_{g}$ is the gas optical path, $C_{g}$ is the concentration, $Q_{g}=C_{g}L_{g}$ is the column density, and the dependence of *a* on wavenumber and temperature has been shown explicitly. If there is more than one absorbing species, τ(ν) is just a product of terms, as in Equation (1), one for each species; if the concentration is not homogeneous, the product aCL is replaced by an integral. Since absorptivities are well-known parameters that can be extracted from spectroscopic databases such as HITRAN [12], a transmittance measurement over a spectral range provides, in principle, an accurate way to identify gases in a sample and to determine their concentrations.

This is the basis of IR absorption spectroscopy, a classical method of analytical chemistry. In its most straightforward laboratory implementation, a gas cell in a spectrophotometer is filled with the sample to be measured, and then with a reference gas without absorption lines in the spectral region of interest (typically $N_{2}$). Transmittance is obtained as the ratio of the two spectra.

**Figure 1:**
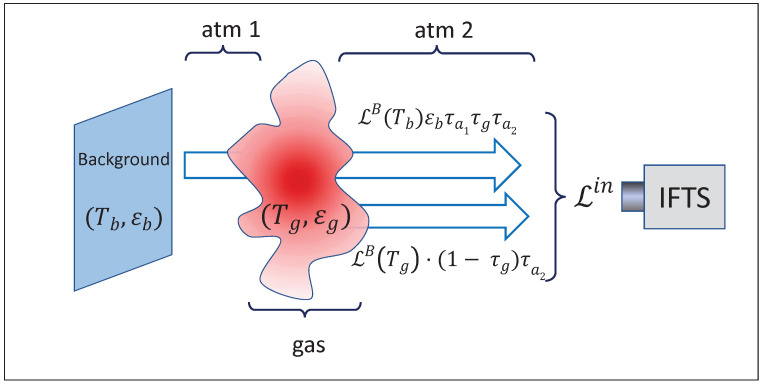
Schematics of the radiative model.

However, the full potential of absorption spectroscopy is displayed in remote measurements. In a typical field measurement with an imaging spectrometer, a gas cloud is observed against a background, and the instrument provides a measurement of the spectral radiance incoming to each pixel. In order to relate this radiance with the gas parameters, a radiative model of the measurement configuration is needed (Figure 1).

### 2.1. Radiative Model

The following simplifying assumptions will be made:

1. The gas is in local thermal equilibrium, so that Boltzmann distribution holds and absorptance α equals emittance ε (Kirchhoff’s Law).
2. The effects of absorption and scattering by particulate matter are negligible.
3. For each pixel, the gas is modeled by a single temperature, and a single value of concentration for each species (these values are considered as line-of-sight averages); therefore, the gas cloud can be characterized by a single transmittance $\tau_{g}$ and emittance $\varepsilon_{g}=\alpha_{g}=1-\tau_{g}$ at each pixel.
4. The background emissivity $\varepsilon_{b}$ is large, so that the reflection of ambient radiation in the background is negligible.
5. The emission of the atmosphere is negligible (i.e., near transparent spectral region, and/or ambient temperature $T_{a}$ much lower than those of gas cloud and background).

With these approximations, the radiance measured by the radiometer can be expressed as:

(2) $$\begin{array}{c} {𝓛}_{m}={𝓛}^{B}\left(T_{b}\right)\cdot \varepsilon_{b}\cdot \tau_{a_{1}}\tau_{g}\tau_{a_{2}}+{𝓛}^{B}\left(T_{g}\right)\cdot \left(1-\tau_{g}\right)\tau_{a_{2}} \end{array}$$

where $\tau_{g}$, $\tau_{a_{1}}$ and $\tau_{a_{2}}$ are, respectively, the transmittances of the gas cloud and the first and second atmospheric paths (atm 1 and atm 2 in Figure 1), ${𝓛}^{B}$ stands for Planck’s blackbody radiance, and $T_{b}$ and $T_{g}$ are, respectively, the temperatures of background and gas cloud.

To obtain a transmittance measurement, a reference spectrum must be measured without gas:

(3) $$\begin{array}{c} {𝓛}_{r}={𝓛}^{B}\left(T_{b}\right)\cdot \varepsilon_{b}\cdot \tau_{a_{1}}\tau_{g_{0}}\tau_{a_{2}} \end{array}$$

where $\tau_{g_{0}}$ stands for the transmittance of the region of atmosphere that was previously occupied by gas cloud; it will be assumed that $\tau_{g_{0}}\approx 1$.

A nominal transmittance is obtained as the ratio:

(4) $$\begin{array}{c} \tau_{nom}\equiv \frac{{𝓛}_{m}}{{𝓛}_{r}}=\tau_{g}+\frac{{𝓛}^{B}\left(T_{g}\right)}{{𝓛}^{B}\left(T_{b}\right)}\cdot \left(1-\tau_{g}\right)\cdot \frac{1}{\varepsilon_{b}\tau_{a_{1}}}\equiv \tau_{g}+\tau^{\prime } \end{array}$$

The positive term $\tau^{\prime }$ is negligible if $\varepsilon_{b}{𝓛}^{B}\left(T_{b}\right)>>{𝓛}^{B}\left(T_{g}\right)$, i.e., when the background is much hotter than the gas; otherwise, the equation can be solved for $\tau_{g}$ if $T_{g}$, $T_{b}$ and $\varepsilon_{b}$ are known (it will be generally assumed that in the spectral region considered, $\tau_{a_{1}}\approx 1$).

### 2.2. Temperature and Column Density Retrieval

Our aim is to obtain the values of gas concentration $C_{g}$ from experimental measurements of ${𝓛}_{r}\left(\nu \right)$ and ${𝓛}_{m}\left(\nu \right)$ but, since only the product CL appears in the equations (cf. Equation (1)), the result can only be the column density $Q_{g}\equiv C_{g}L_{g}$ rather than the concentration $C_{g}$. The amount of gas will be measured, as usual by spectroscopic remote sensing methods, in units of ppm·m (parts per million per meter).

Since absorptivity $a(\nu ,T_{g})$ is a known parameter, the most straightforward method to recover $Q_{g}$ for each gas is to solve Equation (4) for $\tau_{g}$ and then use Lambert–Beer law (1) to obtain $Q_{g}$. However, in many practical cases the gas cloud temperature $T_{g}$ will be unknown, and therefore should also be retrieved simultaneously with $Q_{g}$ from the experimental measurements.

Thus, measurements of ${𝓛}_{r}\left(\nu \right)$ and ${𝓛}_{m}\left(\nu \right)$ over a spectral range rather than at a single ν will be necessary to provide a set of equations, but even so it is not possible to solve Equations (4) and (1) simultaneously for $T_{g}$ and $Q_{g}$, because both parameters are coupled in the Lambert–Beer expression of transmittance (1), where the absorptivity *a* depends on $T_{g}$ in a nontrivial way. Instead, they will be determined by a fitting process: we will calculate theoretical spectra for ${𝓛}_{r}\left(\nu \right)$ and ${𝓛}_{m}\left(\nu \right)$, divide them to obtain a theoretical nominal transmittance $\tau_{nom}^{th}\left(\nu \right)$ and assign to each pixel the column density and temperature values which provide the best fit to the experimental spectra $\tau_{nom}\left(\nu \right)$.

In summary, the final results of our method are a “column density image” and a “temperature image” with values of, respectively, $Q_{g}$ and $T_{g}$ at each point in the field of view, obtained by iteratively fitting the experimental nominal transmittance spectra with theoretical spectra generated according to the radiative model of Figure 1, through the Equations (1)–(4).

### 2.3. Theoretical Spectra and Fitting Procedure

The spectral positions and intensities of the emission/absorption lines have been obtained from the HITRAN database [12]. For methane and nitrous oxide, the HAPI [13] Python-based interface to HITRAN has been used to download the respective absorption coefficients. However, in this free-access database, there is no detailed information about propane. Absorption coefficients for it have been obtained from the absorption cross sections at an atmospheric pressure of 1 atm and three temperatures (278.15 K, 298.15 K and 323.15 K) available on the webpage of HITRAN online [14]. With this information, it is possible to calculate the absorption coefficients by multiplying the cross-section data by the number of molecules per volume unit at ambient conditions.

Theoretical spectra have been generated by summing up the standard linehapes of single absorption lines (“line-by-line method”). The dependence of *a* on temperature, due to variation of absorption cross sections with T, has been fitted by seventh-order polynomial functions with a spectral resolution of 0.01 cm${}^{-1}$[10]. With this parametrization it is easy to construct theoretical transmittance spectra $\tau_{g}^{th}\left(\nu \right)$ for arbitrary values of $T_{g}$ and $Q_{g}$, using Equation (1) and, in turn, theoretical $\tau_{nom}^{th}\left(\nu \right)$ spectra with (2)–(4).

In order to compare these spectra to the measured ones, the effect of finite instrument resolution must be accounted for. In our case, a triangular apodization was used, so that the instrumental lineshape function (ILS) is a squared *sinc* function [15].

However, when calculating the theoretical transmittance spectrum, it is not correct to simply convolve the ideal spectrum with the ILS. The reason is that the experimental nominal transmittance spectrum $\tau_{nom}\left(\nu \right)$ is not measured directly, but rather as a ratio (Equation (4)) of two radiance spectra measured by our instrument, ${𝓛}_{m}$ and ${𝓛}_{r}$. Therefore, the correct theoretical spectrum $\tau_{nom}^{th}\left(\nu \right)$ must be calculated as a ratio of widened radiances:

(5) $$\tau_{nom}^{th}\left(\nu \right)=\frac{\int \left[{𝓛}^{B}\left(\nu^{\prime },T_{b}\right)\cdot \varepsilon_{b}\cdot \tau_{a_{1}}\left(\nu^{\prime }\right)\cdot \tau_{g}\left(\nu^{\prime }\right)\cdot \tau_{a_{2}}\left(\nu^{\prime }\right)+{𝓛}^{B}\left(\nu^{\prime },T_{g}\right)\cdot \left(1-\tau_{g}\left(\nu^{\prime }\right)\right)\cdot \tau_{a_{2}}\left(\nu^{\prime }\right)\right]\cdot ILS\left(\nu -\nu^{\prime }\right)d\nu^{\prime }}{\int {𝓛}^{B}\left(\nu^{\prime },T_{b}\right)\cdot \varepsilon_{b}\cdot \tau_{a_{1}}\left(\nu^{\prime }\right)\cdot \tau_{a_{2}}\left(\nu^{\prime }\right)\cdot ILS\left(\nu -\nu^{\prime }\right)d\nu^{\prime }}$$

where $\tau_{g}\left(\nu \right)$ and $\tau_{a_{1}}\left(\nu \right)$, $\tau_{a_{2}}\left(\nu \right)$ stand for the ideal transmittance spectra of the gas cloud and first and second atmospheric paths, respectively, as provided by HITRAN. They are functions (not explicitly displayed) of the temperatures ($T_{g}$, $T_{a}$) and column densities of the gas cloud ($Q_{g}$) and the atmospheric gases. In this work, it has been assumed that $\tau_{a_{1}}\approx \tau_{a_{2}}\approx 1$, which is a very good approximation for the measurement configuration and the spectral regions involved.

At each pixel, the fitting procedure is as follows (a single gas will be assumed; for each additional gas the procedure is the same but there is an additional unknown value of column density to be determined). We start by assuming a value for the couple $\left(Q_{g},T_{g}\right)$. The theoretical transmittance spectrum $\tau_{nom}^{th}\left(\nu \right)$ is calculated with Equations (1) and (5) at the points of the wavenumber axis of the experimental spectra. The differences with $\tau_{nom}\left(\nu \right)$ for each wavenumber are added up in quadrature to get the sum of squared errors (SSE). The Nelder–Mead minimization algorithm, as implemented in MATLAB software, is used then to find the value of $\left(Q_{g},T_{g}\right)$ for the next iteration, until convergence is reached. This iterative process is repeated for each pixel to obtain the images of column density and temperature.

## 3. Experimental Measurements

**Figure 2:**
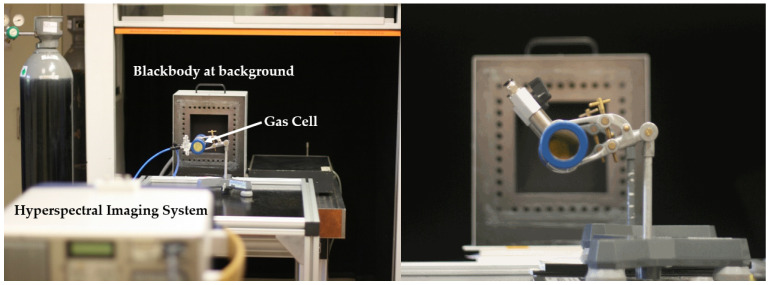
(**Left**) Overall view of the experimental setup. (**Right**) A close-up view of the gas cell without the gas supply tubes.

The experimental setup reproduces the scheme of Figure 1, but with the gas to be measured confined to a gas cell in order to know precisely the optical path (see Figure 2). The three main elements are: a blackbody radiator as a temperature controlled background, a gas cell for the pollutant to be characterized and the imaging Fourier transform spectrometer (IFTS) that captures both spectral and spatial information of the scene.

Specifically, an extended area (15 × 15 cm) blackbody radiator from Santa Barbara Infrared, Inc., with nominal emissivity of 0.9 was placed as uniform background, and a 43 cm long gas cell made of stainless steel with two 38 mm diameter sapphire optical windows was used to enclose the gas under test. This cell has two valves separated by a distance of 20 cm for gas input and output.

The experimental spectra have been acquired with a Telops FIRST-MW Hypercam IFTS [16,17] placed at a distance of two meters from the blackbody radiator, with the 43 cm metallic gas cell in-between. In this instrument, the incoming radiance is modulated by a Michelson interferometer, and then is detected by an InSb 320×256 focal plane array (IFOV = 0.35 mrad), sensitive in the mid-infrared (1850 to 6667 cm${}^{-1}$). Interferograms are acquired for each pixel, which, after processing, can provide spectra with a maximum resolution of 0.25 cm${}^{-1}$.

In order to reduce acquisition time to ≈ 1 min, in this work the spectral resolution of the measurements was set at 1 cm${}^{-1}$ and a spatial sub-windowing of 256 × 160 pixels was used. Integration time was 10 s. Four interferograms were acquired for each measurement, and the dataset was pre-processed by calculating its median and then Fourier-transformed to obtain the radiance spectra. Processing of the interferograms includes triangular apodization, zero-padding to obtain experimental spectra with same wavenumbers as the theoretical ones, as well as off-axis correction [18]. All the processing steps have been described in [10].

Radiance spectra were obtained for both reference (with gas cell filled with N${}_{2}$) and pollutant gas and divided according to Equation (4) to get a nominal transmittance spectrum.

Measurements have been carried out with the gas at ambient temperature and the blackbody background at 350 ${}^{\circ }$C, for methane (CH${}_{4}$), nitrous oxide (N${}_{2}$O) and propane (C${}_{3}$H${}_{8}$) at the concentrations and in the spectral regions detailed in Table 1. The bottles were prepared by the Spanish Metrology Institute (CEM, Centro Español de Metrología), ensuring high accuracy in the concentration values.

## 4. Noise Filtering by Principal Component Analysis

**Figure 3:**
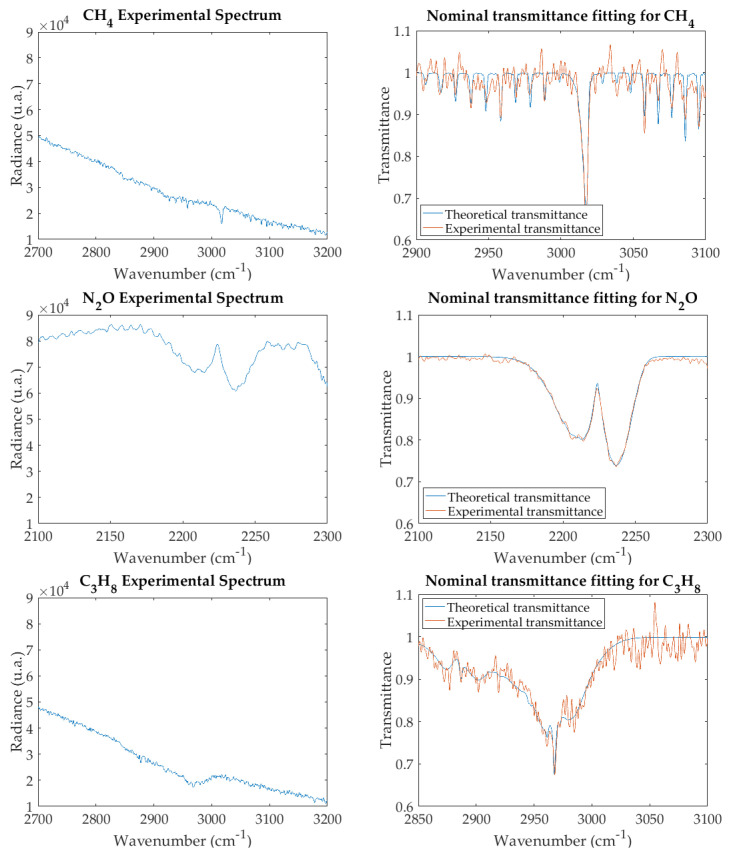
Experimental spectra of air pollutants: radiance (**left**) and nominal transmittance, with best fit (**right**).

Experimental radiance spectra for the three gases studied are shown in the left-hand graphs of Figure 3. These spectra, divided by the reference spectrum obtained with the gas cell full of N${}_{2}$, give the transmittance spectra of the right-hand side. The best fitting by theoretical spectra (achieved with the iterative algorithm as explained in Section 2.3) is also shown.

It is well known that when two noisy spectra are divided, the signal to noise ratio (SNR) decreases greatly. Therefore, it would be very convenient to reduce the noise level of radiance spectra before calculating transmittance. This can be performed by acquiring more interferograms, at the cost of increasing measuring time, or by averaging over neighboring pixels, thus decreasing spatial resolution.

There is, however, a better solution provided by principal components analysis (PCA) [19]. This is a well-known statistical technique used to reduce the dimensionality of sets of multivariate data. If we have *n* measurements, each of *m* variables, the data can be interpreted as a cloud of *n* points in a *m*-dimensional *variable space*. PCA generates a new orthogonal basis in this space, optimally adapted to the data in the sense that (a) its origin coincides with the center of mass of the points and (b) the new (sometimes called “main”) axes are oriented so that the projections of data on them are uncorrelated (i.e., in the new axes, the covariance matrix of the data is diagonal). The unit vectors corresponding to these axes are the eigenvectors of the covariance matrix, and PCA provides them in decreasing order of the associated eigenvalue. This means that the first principal direction is that along which the variance of the data is a maximum; the second principal component is, among the subset of vectors perpendicular to the first, the one whose direction contains the largest variance, and so on. The coordinates of a point in the spectral space with respect to the new basis are called principal components (PCs) or sometimes scores, and are obtained by subtracting the coordinates of the center of mass and then projecting on the basis of eigenvectors.

Since most of the variance of the data is found in the first principal components, a good approximation to the original data set can be made by considering only a small number of principal components, say *p*. This is equivalent to projecting the data set in the *p*-dimensional sub-space built from the first *p* main axes, and achieves a reduction in the dimensionality of the data set from *m* to *p*.

In our case, the original data are the spectra (each one with *m* wavenumbers, m∼15.000 for 1 cm${}^{-1}$ resolution) from a region of *n* pixels corresponding to the gas cell. Since the spectra depend on two variables, T and Q, we can conjecture that the data should have an intrinsic dimensionality close to two. They should all, therefore, lie very close to a surface in the variable space, although this surface will not be a plane, since transmittance is not linear with Q or T. However, if the range of variation of T and Q in the data is relatively small, the corresponding surface region will be approximately flat, so that two principal components should be enough to describe with good approximation all the variability of the original data (p=2). When T and Q have a wider variation, it will be necessary to take p>2, but in any case, the principal components of large order will contain mainly noise. In summary, selecting the subspace spanned by the first major components not only dramatically reduces data volume, but also results in efficient noise filtering [20,21].

**Figure 4:**
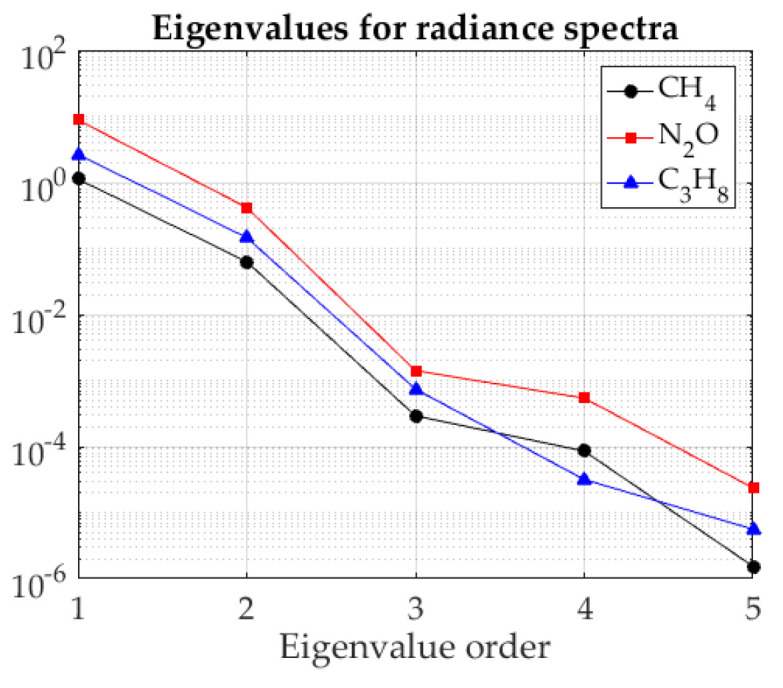
Values of the first 5 eigenvalues for the covariance matrix of the radiance spectra of the three gases studied.

**Figure 5:**
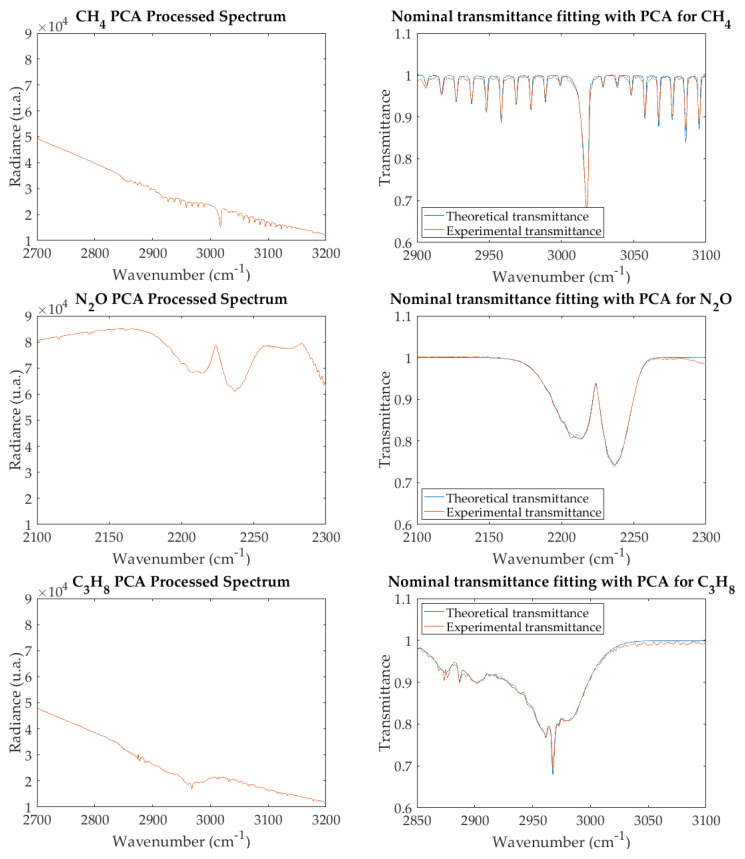
PCA–processed experimental spectra of air pollutants: radiance (**left**) and nominal transmittance, with best fit (**right**).

To apply PCA to our experimental data, a preliminary scene classification is performed by a standard k-means algorithm [22,23] to select the region of the image that corresponds to the gas in the cell. After applying PCA to the radiance spectra in that region, it is found that eigenvalues decrease sharply (Figure 4), so that for all the gases studied the first two account for more than 99.95% of the trace of the covariance matrix (i.e., the total variance of the data). This confirms our conjecture and suggests that a good spectrum reconstruction should be obtained with only two principal components. Indeed, Figure 5 (left-hand side) shows that the reconstructed radiance spectra reproduce with high fidelity the original ones (shown in Figure 3), but with noise filtered out; as expected, the effect is stronger in transmittance (Figure 5, right-hand side). The results of iterative fitting of these spectra are shown also in the right-hand side of Figure 5.

**Figure 6:**
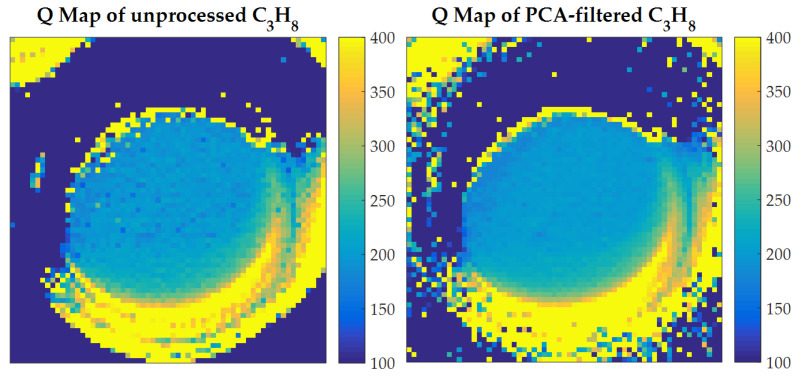
Maps of Q values retrieved by iterative fitting from $\tau_{nom}$, unprocessed (**left**) and PCA-filtered (**right**). The scale is in ppm·m; the size of the field of view is 5.5 cm × 5.5 cm. Retrieved values of Q only have physical meaning in the central round region that corresponds to the gas cell window; it is clear that PCA filtering improves uniformity in that region.

By fitting spectra over the whole field of view of the instrument, a map of retrieved Q is created. Figure 6 compares the C${}_{3}$H${}_{8}$ maps obtained from unprocessed spectra (left) and PCA-filtered spectra (right). As expected, only the round cell window regions have meaningful values, and they are quite similar in both cases, although the PCA-processed map is more uniform.

Retrieved Q values are summarized in Table 2, both for PCA-filtered (Figure 5) and unfiltered spectra (Figure 3). Values are the mean ± the standard deviation in a square of 7×7 pixels at the center of the gas cell. Signal to noise ratios measured in dB are also tabulated. PCA increases SNR in all cases, and the effect is larger the noisier is the original spectrum: the dB value is multiplied by 3.2 for CH${}_{4}$, by 2.1 for C${}_{3}$H${}_{8}$, and by 1.1 for N${}_{2}$O. It must be pointed out that this improvement does not come at the expense of spatial resolution (which is not degraded) or acquisition time (which is not increased), since no spatial or time averaging is involved.

Comparison of the retrieved Q values with the nominal ones gives relative errors of −2.6% for CH${}_{4}$, +4.8% for N${}_{2}$O and −9.2% for C${}_{3}$H${}_{8}$ for non-PCA-processed spectra and similar values for the PCA-processed, except for a slightly better value for C${}_{3}$H${}_{8}$ (relative error −7.1%). These results, however, do not mean that PCA does not improve the measurement of Q. Since they have been obtained by spatially averaging over a uniform region, the most relevant parameter here is standard deviation, which is much smaller for PCA-filtered spectra. The conclusion to be extracted is that the main effect of PCA processing has been to improve the precision of retrieval rather than its accuracy.

Regarding the retrieved temperatures, for a room $T_{g}\approx 302$ K, results for CH${}_{4}$, N${}_{2}$O and C${}_{3}$H${}_{8}$ were, respectively, 310.6±25.6 K, 305.0±2.2 K and 312.6±16.7 K for non-PCA-processed spectra, and 306.7±2.4 K, 305.4±1.4 K and 312.5±8.7 K for the PCA-processed. These values show a similar behavior to those of Q: PCA processing has only improved slightly the value of T for CH${}_{4}$ but has achieved an important reduction in standard deviations, i.e., gives better results regarding uniformity.

## 5. Dimensionality Reduction by Principal Component Analysis

Up to now, PCA has been applied to a datacube of experimental nominal transmittance spectra and has been used only to filter out noise in those spectra by reconstructing them with a small number *p* of PCs (in the cases studied here, p=2). $Q_{g}$ and $T_{g}$ have been retrieved by iterative fitting of the filtered spectra.

However, since filtered spectra are characterized by only p∼2 PCs, it seems that it is very inefficient to perform fitting in the full spectral space (where our objects are vectors of m∼15.000 components) instead of the subspace spanned by the relevant eigenvectors (where our objects are vectors of *p* components; we call this space “PC space”).

The reason for this procedure is that simulation of spectra is based on the physics of absorption/emission and generates them line by line. So the spectra on which the iterative algorithm operates belong to the spectral space and have *m* components. If we want to operate in the PC space, they could be projected onto the *p* first eigenvectors obtained with PCA; then, the error between experiment and simulation could be calculated for the PCs. However, the bulk of the computation time is spent on the line-by-line simulation of the spectra and, once they are calculated, calculation of error is relatively straightforward. Thus, there is no appreciable efficiency gain in projecting the spectra on eigenvectors during iterative fitting and calculate errors in the PC space.

### 5.1. Retrieval by Search on Pre-Calculated Datacube

The previous observation underlines that the bottleneck of the retrieval process is the iterative generation of simulated spectra during fitting. Thus, a great improvement in efficiency could, in principle, be achieved by avoiding that process. This can be achieved if spectra are pre-calculated, as follows:

1. For a specific scene, a matrix of ($T_{g},Q_{g}$) values can be defined, such that the ranges of $T_{g}$ and $Q_{g}$ cover the expected values in the scene. Nominal transmittance spectra $\tau_{nom}\left(\nu ,Q_{g},T_{g}\right)$ can be calculated for all the ($T_{g}$, *Q*) values of the matrix (for a given background temperature $T_{b}$). A  simulated spectra datacube is thus obtained.
2. A experimental spectrum can now be compared to all the spectra of this datacube; the ($T_{g},Q_{g}$) couple retrieved is the one that gives the smaller error (this can be measured as the sum of squared errors, SSE, or as the absolute error).

To test this procedure, simulated spectra datacubes with a spectral resolution of 1 cm${}^{-1}$ and $T_{b}=350{\;}^{\circ }$C were calculated for each of the three pollutant gases studied. Gas temperatures varied between $0{\;}^{\circ }$C $\leq T_{g}<69{\;}^{\circ }$C with a step $\Delta T_{g}=1{\;}^{\circ }$C, and the range of column densities was 70 ppm·m, centered for each gas at its expected column density, with $\Delta Q_{g}=1$ ppm.

Results are shown in Table 3, under the heading SSD (simulated spectra datacube). Comparison with nominal values gives relative errors of −7.8% for CH${}_{4}$, +5.1% for N${}_{2}$O and −7.4% for C${}_{3}$H${}_{8}$, similar to those of the iterative fitting method except for a larger value in CH${}_{4}$. Standard deviations are of the same order of those obtained previously with PCA-processed spectra.

Generation of each simulated spectra datacube took 23.2 s of CPU time in an Intel i7 processor based computer at 3.2 GHz, with six cores and 64 GB of RAM. Then, the realization of a column density map over a region of 70 × 70 pixels took 5630 s of CPU time. This result was unexpected, since it is longer than the 1460 s of CPU time for the same task if completed by pixel-by-pixel iterative fitting.

The explanation is that in order to find the ($T_{g},Q_{g}$) couple at each pixel an exhaustive search was used, i.e., the SSE was calculated between the experimental spectrum and *all* the spectra in the simulated datacube. This is a very inefficient strategy, and time can be reduced at least by an order of magnitude if a gradient search algorithm is used. Clearly, time will also be shorter if the simulated spectra datacube is made smaller, either by increasing the steps ($\Delta T_{g},\Delta Q_{g}$) or by reducing the range of ($T_{g},Q_{g}$). No attempt of improvement along these lines has been made, however, since the approach based in PCA described in the following section is much more powerful.

### 5.2. Simulated PC Datacube

The retrieval strategy just described above compares experimental spectra as measured (i.e., in the spectral space) with the simulated ones. However, it can be enhanced by the use of principal components to make it faster.

If a PCA is performed on the simulated spectra datacube, its z dimension can be drastically reduced. The datacube thus obtained will be called the *simulated PC datacube*. Now, the number *p* of PCs needed may be larger than 2, since spectra in the simulated datacube have a larger variability than those of gas cell, because of the much wider interval of temperatures and column densities involved. However, the absence of noise reduces the variance of the simulated spectra, and, in our case, p=2 is still enough to account for more than 99.95% of the total variance.

Now, to retrieve the values of $T_{g}$ and $Q_{g}$ for a pixel, the experimental spectrum is projected onto the first *p* eigenvectors of the simulated spectra datacube, in order to obtain its PCs (scores), and these *p* numbers are compared by a simple exhaustive search with those in the simulated PC datacube to find the ($T_{g},Q_{g}$) couple with optimal agreement. It is important, however, not to make the direct comparison of the scores, but rather to multiply them by the magnitude of the corresponding eigenvector so as to to calculate correctly the distance between the experimental and the simulated spectra in the PC space.

Retrieval of Q and T is dramatically faster with this procedure. Generation of the simulated PC datacube from the simulated spectra datacube took 2.3 s of CPU. Then, creation of a map of Q over the same 70 × 70 region as above took only 1.0 s of CPU.

Results are shown in Table 3, under the heading SPCD (simulated PC datacube). Relative errors as compared to nominal values are now much smaller than previously: −1.9% for CH${}_{4}$, +2.7% for N${}_{2}$O and 1.4% for C${}_{3}$H${}_{8}$. Standard deviations are of the same order, being somewhat smaller for CH${}_{4}$ and larger for C${}_{3}$H${}_{8}$.

Retrieved temperatures are also more accurate, and nearly identical for the three gases: 305.1±2.7 K for CH${}_{4}$, 305.7±1.5 K for N${}_{2}$O and 304.7±5.1 K for C${}_{3}$H${}_{8}$.

A point worth noting is that, since this approach is based on a PCA performed on simulated spectra rather than on experimental ones, it can be applied as well to non-imaging spectrometers.

### 5.3. Retrieval of Q and T by Polynomial Fitting of Principal Components

One appealing aspect of the approach developed here is that the temperature and column density of the pollutant gas can be retrieved even without the ability to perform the complex process of spectrum simulation explained in Section 2.3. Rather, for a specific measurement conditions, with known $T_{b}$ and expected ranges of $T_{g}$ and $Q_{g}$, the user can be provided with the mean spectrum and the first *p* eigenvectors of the relevant simulated spectra datacube. Then, the components on the PC base of the experimental spectra can be written by subtracting the mean spectrum and projecting onto the eigenvectors.

In the previous section, $T_{g}$ and $Q_{g}$ for a pixel were obtained by an exhaustive search in the simulated PC datacube, to find the best agreement with those components. However, this can be further simplified for the user if explicit functions can be found, $T_{g}=T_{g}\left(PC_{1},\cdots PC_{p}\right)$ and $Q=Q(PC_{1},\cdots PC_{p})$, that fit the dependence of $T_{g}$ and $Q_{g}$ from the PCs, as defined in the simulated PC datacube.

**Figure 7:**
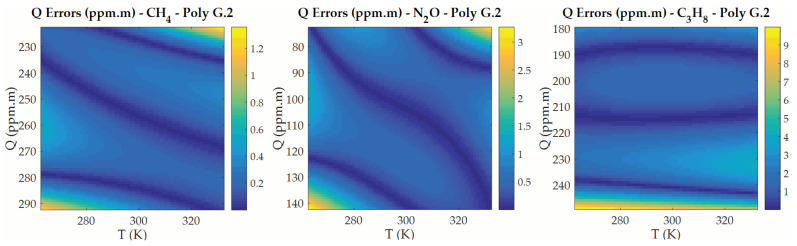
Absolute errors in the Q values obtained as second-degree polynomial functions of $T_{g}$ (horizontal axis) and $Q_{g}$ (vertical axis) for each of the gases studied. Errors are very small except for the cases when (T, Q) values are either very large or very small (for CH${}_{4}$ and N${}_{2}$O) and only for the very small values of Q (for C${}_{3}$H${}_{8}$).

This has been perfomed for the three gases under study in this work, using the function package polyfitn available for use in MATLAB. It has been found that second-degree polynomial functions can provide values for $T_{g}$ and $Q_{g}$ as functions of $\left(PC_{1},PC_{2}\right)$, with very small errors. As an example (Figure 7), the error of the $Q_{g}$ values furnished by the polynomial function is smaller than ±0.7 ppm·m for CH${}_{4}$, ±1.7 ppm·m for N${}_{2}$O, and ±5.5 ppm·m for C${}_{3}$H${}_{8}$ for most of the (T, Q) values of the pre-calculated datacube.

## 6. Summary and Conclusions

The only way to improve signal-to-noise ratio (SNR) in a specific measurement condition with a non-imaging spectrometer was to average many spectra. In imaging spectrometers, averaging can be made over neighbouring pixels. In both cases, SNR improvement comes at a cost: time averaging degrades time resolution, and spatial averaging degrades spatial resolution.

Imaging spectroscopy, however, makes possible a better strategy: to apply principal component analysis to the datacube of experimental radiance spectra, and then reconstruct the spectra using only a reduced number of principal components. The reconstructed spectra have noise filtered out without losing spatial resolution.

In this work, this strategy has been applied to optimize measurements of column density (Q, concentration·path product) and temperature (T) of pollutant gases, specifically, methane, nitrous oxide, and propane.

A radiometric model that takes into account radiation emission and absorption, as well as instrumental lineshape, has been defined and applied to generate line-by-line theoretical spectra using the spectroscopic parameters of the HITRAN database. These spectra are compared to experimental spectra measured for the pollutant gases in order to retrieve their Q and T values. With an extended blackbody as background, two radiance spectra are acquired for each pixel: one with the gas cell full of pollutant at the prescribed concentration, the other with nitrogen as a reference, non-absorbing gas.

After PCA-processing, the increase in SNR, measured in dB, has been ×1.1 for N${}_{2}$O, ×2.1 for C${}_{3}$H${}_{8}$, and ×3.2 for CH${}_{4}$. These PCA-processed spectra have been used to obtain the nominal transmittance spectra whose comparison to theoretical spectra provides the retrieved Q and T values.

The more straightforward way to make that comparison is to generate theoretical spectra, and to compare them iteratively, wavenumber by wavenumber, to the experimental ones until the sum of squared errors is minimized. It has been found that the retrieved values of Q had a typical error of ∼7% both for unprocessed and PCA-processed spectra, although the latter provided better uniformity, with smaller standard deviations.

The strategy just described is, however, very slow and computing-intensive. PCA can be used also to speed up this process if the theoretical nominal transmittance spectra are pre-calculated for a range of T and Q appropriate to the expected values of the gas, and then PCA is applied to this *simulated spectra datacube*. Then, the comparison between experimental and theoretical spectra can be made in the PC space, whose dimension is drastically smaller than that of the spectra (in our case, two PCs versus ∼ 15.000 wavenumbers). Thus, a very significant reduction in calculation time (a factor larger than one thousand) is achieved. Accuracy of the retrieved Q and T values is also substantially improved: typical errors in retrieved Q values have been found to be ∼2%.

This procedure can be further simplified when the measurement conditions are repetitive, with known background temperature and gas T and Q within specific ranges. The user can be supplied with the results of the PCA applied to the relevant simulated datacube (mean spectrum and first eigenvectors), and can use them to obtain the first PCs of the experimental spectra. Then, if the ranges of T and Q are not too wide (e.g., $70{\;}^{\circ }$C and 70 ppm·m in this work), explicit polynomic functions can be fitted to the simulated PC datacube that directly provides Q and T as functions of the first two PCs of the spectra. In this approach, the user only needs to measure the experimental nominal transmittance spectra, with no need to calculate simulated spectra or perform iterative fittings, and without significant loss of accuracy in the results.

## Figures and Tables

**Table 1:** Air pollutants under test.

Pollutant Gas	Concentration (ppm)	Column Density (ppm·m)	Bandwidth (cm${}^{-1}$)
CH${}_{4}$	600	258	2700–3200
N${}_{2}$O	250	107.5	2100–2300
C${}_{3}$H${}_{8}$	500	215	2700–3200

**Table 2:** Column density values retrieved and signal to noise ratio for air pollutants in a 7×7 square at the center of the gas cell. Values obtained by iterative search using as-measured experimental spectra and PCA-processed experimental spectra.

Gas	Nominal Q (ppm·m)	Retrieved Q w/o PCA (ppm·m)	Retrieved Q with PCA (ppm·m)	SNR w/o PCA (dB)	SNR with PCA (dB)
CH${}_{4}$	258	251.2±33.7	250.0±9.8	5.5±0.8	17.7±0.1
N${}_{2}$O	107.5	112.7±1.6	112.3±1.4	24.2±0.8	26.1±3.3
C${}_{3}$H${}_{8}$	215	195.2±8.6	199.7±3.8	12.6±0.5	26.1±1.1

**Table 3:** Column density retrieved for air pollutants in a 7×7 square at the center of the gas cell. Values obtained by search in simulated spectra datacube (SSD) and in simulated PC datacube (SPCD).

Gas	Nominal Q (ppm)	Retrieved Q SSD (ppm)	Retrieved Q SPCD (ppm)
CH${}_{4}$	258	237.9±11.7	253.2±7.6
N${}_{2}$O	107.5	113±1.4	110.4± 3.4
C${}_{3}$H${}_{8}$	215	199.1±3.3	218.1± 7.4

## References

[1] Héroux M.-E., Anderson H.R., Atkinson R., Brunekreef B., Cohen A., Forastiere F., Hurley F., Katsouyanni K., Krewski D., Krzyzanowski M. (2015). Quantifying the healthimpacts of ambient air pollutants: Recommendations of a who/europe project. Int. J. Public Healt..

[2] IMPRESS 2: Metrology for Air Pollutant Emissions. http://empir.npl.co.uk/impress/.

[3] Hodgkinson J., Tatam R.P. (2012). Optical gas sensing: A review. Meas. Sci. Technol..

[4] Prengle H.W., Morgan C.A., Fang C.-S., Huang L.-K., Campani P., Wu W.W. (1973). Infrared remote sensing and determination of pollutants in gas plumes. Environ. Sci. Technol..

[5] Herget W.F. (1982). Remote and cross-stack measurement of stack gas concentrations using a mobile FT-IR system. Appl. Opt..

[6] Wormhoudt J. (1985). Infrared Methods for Gaseous Measurements: Theory and Practice.

[7] Manolakis D.G., Lockwood R.B., Cooley T.W. (2016). Hyperspectral Imaging Remote Sensing: Physics, Sensors, and Algorithms.

[8] Gross K.C., Bradley K.C., Perram G.P. (2010). Remote Identification and Quantification of Industrial Smokestack Effluents via Imaging Fourier-Transform Spectroscopy. Environ. Sci. Technol..

[9] Harley J.L., Gross K.C. (2011). Remote quantification of smokestack effluent mass flow rates using imaging Fourier transform spectrometry. Proc. SPIE.

[10] Rodríguez-Conejo M.A., Meléndez J. (2015). Hyperspectral quantitative imaging of gas sources in the mid-infrared. Appl. Opt..

[11] Gålfalk M., Olofsson G., Bastviken D. (2017). Approaches for hyperspectral remote flux quantification and visualization of GHGs in the environment. Remote. Sens. Environ..

[12] Gordon I.E., Rothman L.S., Hill C., Kochanov R.V., Tan Y., Bernath P.F., Birk M., Boudon V., Campargue A., Chance K.V. (2017). The HITRAN2016 molecular spectroscopic database. J. Quant. Spectrosc. Radiat. Transf..

[13] Kochanov R.V., Gordon I.E., Rothman L.S., Wcisło P., Hill C., Wilzewski J.S. (2016). HITRAN Application Programming Interface (HAPI): A comprehensive approach to working with spectroscopic data. J. Quant. Spectrosc. Radiat. Transf..

[14] HITRANonline. https://hitran.org/.

[15] Griffiths P.R., De Haseth J.A. (2007). Fourier Transform Infrared Spectrometry.

[16] Chamberland M., Farley V., Vallieres A., Villemaire A., Belhumeur L., Giroux J., Legault J.-F. (2005). High-performance fieldportable imaging radiometric spectrometer technology for hyperspectral imaging applications. Proc. SPIE.

[17] Gagnon J., Habte Z., George J., Farley V., Tremblay P., Chamberland M., Romano J., Rosario D. (2010). Hyper-Cam automated calibration method for continuous hyperspectral imaging measurements. Proc. SPIE.

[18] Gross K.C., Tremblay P., Bradley K.C., Chamberland M., Farley V., Perram G.P. (2010). Instrument calibration and lineshape modeling for ultraspectral imagery measurements of industrial smokestack emissions. Proc. SPIE.

[19] Shlens J. (2014). A tutorial on principal component analysis. arXiv.

[20] Natarajan B., Konstantinides K., Herley C. (1998). Occam filters for stochastic sources with application to digital images. IEEE Trans. Signal Process..

[21] Antonelli P., Revercomb H.E., Sromovsky L.A., Smith W.L., Knuteson R.O., Tobin D.C., Garcia R.K., Howell H.B., Huang H.-L., Best F.A. (2004). A principal component noise filter for high spectral resolution infrared measurements. J. Geophys. Res. Atmos..

[22] Kanungo T., Mount D.M., Netanyahu N.S., Piatko C.D., Silverman R., Wu A.Y. (2002). An efficient k-means clustering algorithm: Analysis and implementation. IEEE Trans. Pattern Anal. Mach. Intell..

[23] Duda R.O., Hart P.E., Stork D.G. (2000). Pattern Classification.

